# Role of α2-Adrenoceptors in Hypertension: Focus on Renal Sympathetic Neurotransmitter Release, Inflammation, and Sodium Homeostasis

**DOI:** 10.3389/fphys.2020.566871

**Published:** 2020-11-09

**Authors:** Lydia Hering, Masudur Rahman, Sebastian A. Potthoff, Lars C. Rump, Johannes Stegbauer

**Affiliations:** Department of Nephrology, Medical Faculty, University Hospital Düsseldorf, Heinrich-Heine-University Düsseldorf, Düsseldorf, Germany

**Keywords:** renal sympathetic nervous system, hypertension, α2-adrenoceptors, sodium transporters, renal vasculature resistance, renal sympathetic neurotransmission, immune cells, macrophages

## Abstract

The kidney is extensively innervated by sympathetic nerves playing an important role in the regulation of blood pressure homeostasis. Sympathetic nerve activity is ultimately controlled by the central nervous system (CNS). Norepinephrine, the main sympathetic neurotransmitter, is released at prejunctional neuroeffector junctions in the kidney and modulates renin release, renal vascular resistance, sodium and water handling, and immune cell response. Under physiological conditions, renal sympathetic nerve activity (RSNA) is modulated by peripheral mechanisms such as the renorenal reflex, a complex interaction between efferent sympathetic nerves, central mechanism, and afferent sensory nerves. RSNA is increased in hypertension and, therefore, critical for the perpetuation of hypertension and the development of hypertensive kidney disease. Renal sympathetic neurotransmission is not only regulated by RSNA but also by prejunctional α2-adrenoceptors. Prejunctional α2-adrenoceptors serve as autoreceptors which, when activated by norepinephrine, inhibit the subsequent release of norepinephrine induced by a sympathetic nerve impulse. Deletion of α2-adrenoceptors aggravates hypertension ultimately by modulating renal pressor response and sodium handling. α2-adrenoceptors are also expressed in the vasculature, renal tubules, and immune cells and exert thereby effects related to vascular tone, sodium excretion, and inflammation. In the present review, we highlight the role of α2-adrenoceptors on renal sympathetic neurotransmission and its impact on hypertension. Moreover, we focus on physiological and pathophysiological functions mediated by non-adrenergic α2-adrenoceptors. In detail, we discuss the effects of sympathetic norepinephrine release and α2-adrenoceptor activation on renal sodium transporters, on renal vascular tone, and on immune cells in the context of hypertension and kidney disease.

## Introduction

Hypertension is the second common cause for end stage renal disease (ESRD) and one of the major risk factors for morbidity and mortality worldwide (GBD 2016 Risk Factors Collaborators, 2017). The kidney is a master regulator of blood pressure homeostasis by regulating vascular tone, as well as sodium and water handling. Renal dysfunction, such as an increase in sodium and water retention, renin release, or renal vascular resistance, causes hypertension and, subsequently in the long-term run chronic kidney damage. The kidney is extensively innervated by sympathetic nerves, which are playing an important role in the regulation of blood pressure homeostasis (Dibona and Kopp, 1997; Dibona, 2000; Grassi et al., 2015). Renal nerves follow the renal arteries and innervate not only the vasculature but also the juxtaglomerular apparatus and the basement membrane of epithelial cells within the nephron. Therefore, it is not surprising that the main neurotransmitter neuropeptide Y (NPY), ATP, and norepinephrine, released at neuroeffector junctions in the kidney, mediate several physiological effects within the kidney. Sympathetic norepinephrine release induces renal vasoconstriction and stimulates renin release as well as tubular sodium and water reabsorption in the kidney. In hypertensive patients, renal sympathetic nerve activity (RSNA) is increased (Schlaich et al., 2009; Grassi et al., 2015). Thus, increased RSNA results in a reduction of renal blood flow and glomerular filtration rate (GFR), an increase in renal vascular resistance and tubular sodium and water reabsorption, and an increased release of renin, contributing to the development and maintenance of hypertension. Studies performed in patients with therapy resistant hypertension show a robust reduction in blood pressure after renal denervation, highlighting a critical crosstalk between the sympathetic nervous system and the kidney in hypertension (Schlaich et al., 2009; Kandzari et al., 2018; Vonend et al., 2018; Steinberg et al., 2020). In addition, evidence emerges that hypertension is at least in part an immune-mediated inflammatory disease. In this regard, several studies have shown a close interaction between the sympathetic nervous system and immune cell response in hypertension. Thus, reduction in RSNA by renal denervation reduces pro-inflammatory markers and immune cell migration in humans and mice (Xiao et al., 2015; Zaldivia et al., 2017).

**Figure 1:**
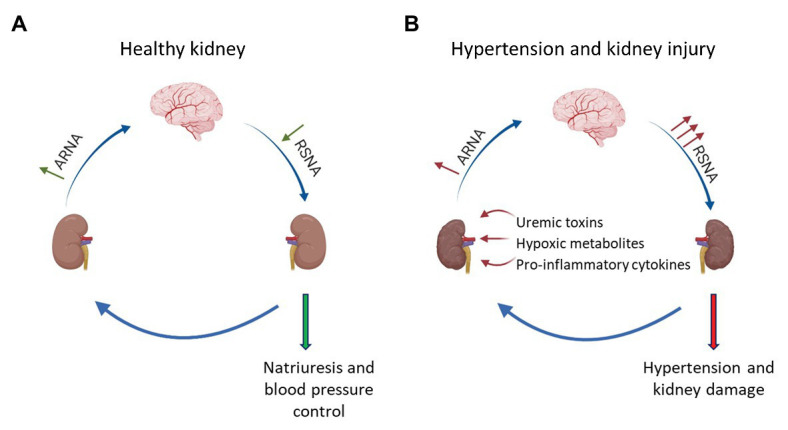
Interaction between afferent renal nerves and renal sympathetic nerve activity in healthy and injured kidneys. **(A)** In healthy kidneys, increased renal sympathetic nerve activity (RSNA) leads to an activation of α1-adrenoceptors expressed in the renal pelvis, which increases afferent renal nerve activity (ARNA). Subsequently, inhibitory neurons within the brainstem and hypothalamus decrease RSNA *via* a negative feedback mechanism leading to natriuresis. **(B)** In hypertension or kidney injury, accumulation of pro-inflammatory cytokines, uremic toxins, or ischemic metabolites activate afferent renal nerves. Under these conditions, the negative feedback mechanism is dysregulated and increased ARNA results in a further activation of renal sympathetic nerves causing the progression of hypertension and hypertensive kidney disease.

**Figure 2:**
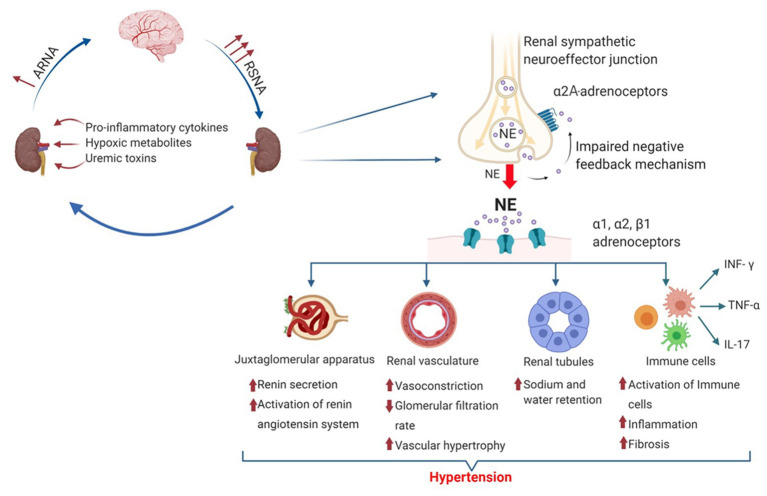
Mechanisms causing hypertension by increased renal sympathetic norepinephrine release. The amount of norepinephrine released by prejunctional renal sympathetic nerves is controlled by RSNA and prejunctional α2A-adrenoceptors. Norepinephrine activates different adrenoceptors and exerts various physiological and pathophysiological effects leading to hypertension and chronic kidney disease (CKD). Thereby, norepinephrine activates the renin-angiotensin system (RAS) by stimulating renin release from the juxtaglomerular apparatus *via* β2-adrenergic receptor activation. Activation of α1- and α2-adrenoceptors causes vasoconstriction and increases renal vascular resistance, leading to vascular hypertrophy and a reduced glomerular filtration rate (GFR). In renal tubules, NE induced α1-, α2-, and β1-adrenergic receptor activation modulates the activity of different sodium transporters such as sodium hydrogen exchanger 3 (NHE3), Na^+^-Cl^−^ Co-transporter (NCC), and epithelial sodium channel (ENaC) leading to decreased sodium excretion. In addition, norepinephrine modulates immune cell function and phenotype, leading to an increased infiltration into the kidney and an increased release of various pro-inflammatory cytokines such as interferon gamma (INF-γ), tumor necrosis factor alpha (TNF-α), and interleukin-17 (IL-17) aggravating the development of hypertension and renal fibrosis.

To understand the role of RSNA in the development of hypertensive kidney disease, it is essential to know how RSNA affects mechanisms in the kidney controlling blood pressure homeostasis. The amount of neurotransmitter released from renal prejunctional nerve endings is not only controlled by the RSNA but also by prejunctional alpha2-adrenergic receptors (α2-adrenoceptors). Prejunctional α2-adrenoceptors serve as autoreceptors which, when activated by norepinephrine released from sympathetic nerve endings, inhibit the subsequent release of norepinephrine induced by a sympathetic nerve impulse (Figures 1, 2). Recent studies have highlighted the critical role of α2-adrenoceptors in the development of hypertension and kidney disease (Kim and Padanilam, 2013; Hering et al., 2020). However, α2-adrenergic receptors are not only expressed prejunctional on sympathetic nerves but also on non-adrenergic cells like immune cells, vascular smooth muscle cells (VSMCs), and renal epithelial cells. Activation of α2-adrenoceptors on these cells mediates a variety of effects, including inflammatory and fibrotic responses (Kim and Padanilam, 2013, 2015) and changes in renal vasoconstriction and VSMC turnover (Bohmann et al., 1995; Jackson et al., 2001, 2005), as well as altering sodium balance (Nord et al., 1987; Mansley et al., 2015) which may also influence blood pressure and kidney damage.

In the present review, we will highlight the role of α2-adrenoceptors on RSNA and its impact on hypertension. Moreover, we will focus on physiological and pathophysiological effects which were mediated by non-adrenergic α2-adrenoceptors with special respect to renal epithelial cells and immune cells. Most of the mechanisms described in the present review are based on animal studies.

## Central Effects and Polymorphism of α2-Adrenoceptors in Hypertension

While this review focuses on physiological and pathophysiological effects mediated by the prejunctional and non-adrenergic α2-adrenoceptors expressed in the kidney and on immune cells, it should be noted that all three α2-adrenoceptor subtypes are widely distributed throughout the central nervous system (CNS) that is ultimately regulating sympathetic nerve activity. Central acting α2-adrenoceptor agonists such as clonidine, guanabenz, and moxonidine are effective in the treatment of hypertension (Kanagy, 2005). These sympatholytic agents cross the blood-brain barrier and interact with central α2-adrenoceptors, leading to a reduction in sympathetic nerve activity and an increase in vagal activity. This change in sympathetic tone causes lower cardiac output and heart rate, reduced renin release, and subsequently a reduction in vascular resistance leading to blood pressure reduction (Dibona and Kopp, 1997; Hein, 2006; Schlaich et al., 2012; Sata et al., 2018). In addition, sympatholytic treatment induced by moxonidine has been shown to attenuate the progression of chronic kidney disease (CKD) in hypertensive patients and rats with advanced renal failure (Amann et al., 2000; Vonend et al., 2003). Whether these effects are in part mediated by non-central effects of α2-adrencoeptor activation is still not fully understood.

Several studies have investigated the association of hypertension and polymorphism of human ADRA2 gene. Genes of α2-adrenocepotor subtypes ADRA2A (α2A-adrenoceptors), ADRA2B (α2B-adrenoceptors), and ADRA2C (α2C-adrenoceptors) are located on chromosomes 10, 2, and 4, respectively. The ADRA2A 1780 C > T (rs553668) genotype is associated with an exercise-dependent aggravation of systolic as well as diastolic blood pressures in women (Nunes et al., 2014). In addition, the described polymorphism is associated with increased platelet aggregation and a marked decrease in sodium excretion. Both findings are common in essential hypertension (Freeman et al., 1995). Moreover, the −1291 C > G (rs1800544) substitution in the ADRA2A promoter region is responsible for reduced presynaptic autoinhibition of α2A-adrenoceptors, resulting in excessive norepinephrine concentration and, therefore, in an increased vascular resistance (Kelsey et al., 2012). Deletion polymorphisms or different variants of ADRA2B and ADRA2C are known to be related to endothelial dysfunction, heart failure, and hypertension (Heinonen et al., 2002; Small et al., 2002; von Wowern et al., 2004; Matsunaga et al., 2007).

In conclusion, there is substantial evidence that genetic variability in ADRA2A and ADRA2B genes influences α2-adrenoceptor function, leading to hypertension due to modulating vascular resistance, endothelial function, and sodium homeostasis in different cohorts.

## Mechanisms Regulating Renal Sympathetic Nerve Activity

The regulation of RSNA is complex and involves central and peripheral mechanism. In general, the nerve activity of sympathetic premotor nuclei in the brainstem and hypothalamus [the rostral ventrolateral medulla (RVLM) and rostral ventromedial medulla (RVMM) as well as the paraventricular nucleus (PVN)] regulates RSNA. While the exact regulatory system of these central mechanisms is not the focus of the current review (Zheng and Patel, 2017), it is noteworthy that central nerve activity in the RVLM, RVMM, and PVN is modulated by neurotransmitters, local factors such as reactive oxygen species, cytokines, and angiotensin II (Ang II), as well as mechano- and chemo-sensitive renal afferent nerves which project to the RVLM *via* the nucleus tractus solitarius (NTS) and PVN. According to physiological or pathophysiological conditions, afferent renal nerve activity (ARNA) can either activate or inhibit sympathetic premotor activity and thereby RSNA *via* a positive or negative feedback mechanism (Dibona, 2000; Pyner, 2014; Zheng and Patel, 2017).

Under physiological conditions, RSNA is controlled by the renorenal reflex, which is considered as a negative feedback loop to maintain efferent RSNA (ERSNA) at low-levels, and thereby controlling natriuresis and blood pressure. This interaction between efferent sympathetic nerves and afferent sensory nerves is complex. Increased RSNA increases ARNA by activating mechanoceptors and chemoceptors, which in turn lowers efferent RSNA *via* inhibitory neurons which project to the RVLM (Dibona and Kopp, 1997; Kopp et al., 2007, 2011a). Norepinephrine acting on adrenoceptors located in the renal pelvis mediates the ERSNA-ARNA interaction. Activation of α1-adrenoceptors leads to an increase in ARNA whereas activation of α2-adrenoceptors decreases ARNA (Kopp et al., 2007). In this regard, Kopp et al. (2011b) showed that low sodium diet reduces ARNA *via* α2-adrenoceptor activation leading to an increase in RSNA and consequently to sodium reabsorption. In contrast, in spontaneous hypertensive rats (SHRs), this mechanism seems to be dysregulated. Thus, ARNA is reduced in SHRs due to an overactivation of α2-adrenoceptors in renal pelvic tissue. These studies suggest a direct role of renal pelvic α2-adrenoceptors in decreasing the responsiveness of ARNA to increased RSNA and thereby in the development of hypertension (Kopp et al., 2007, 2011a). In contrast, a recent study performed in α2A-adrenoceptor deficient mice showed that deletion of α2A-adrenoceptors accelerates Ang II-dependent hypertension rather than decreases blood pressure (Hering et al., 2020). This study suggests that the renorenal reflex mediated by α2-adrenoceptors is dysregulated or does not seem to play an important role in this experimental model of hypertension.

In hypertension as well as acute or chronic kidney damage, stimulation of renal nociceptive afferent nerves mediate an increase in sympathetic nerve activity leading to a further activation of RSNA and subsequently to a progression of hypertension and hypertensive kidney disease. Thus, several other factors such as pro-inflammatory cytokines (Banek et al., 2019), uremic toxins (Campese and Kogosov, 1995), and hypoxia (Dibona and Kopp, 1997; Soukhova-O'hare et al., 2006; Saha et al., 2019) can activate the chemo- and mechano-sensitive afferent nerves leading to an increased RSNA. Activation of afferent nerves under pathophysiological conditions such as acute kidney injury induced by a phenol injection into the kidney causes hypertension by increasing RSNA *via* afferent renal nerve stimulation (Ye et al., 2002a,b; Leong et al., 2006). The importance of an increased ARNA in the development and maintenance of hypertension in acute kidney disease or CKD is supported by several studies. For instance, in hypertensive rats treated with deoxycorticosterone acetate (DOCA) salt, increased ARNA seems to perpetuate hypertension as selective ablation of the afferent renal nerves reduces blood pressure (Banek et al., 2016). Additionally, in patients with kidney failure treated with dialysis, increased sympathetic nerve activity and thereby hypertension could only be reduced by a bilateral removal of the kidneys (Converse et al., 1992).

In summary, renal pelvic α1- and α2-adrenoceptors affect the renorenal reflex that regulates RSNA activity *via* ARNA under physiological conditions. In hypertension or kidney injury, ARNA is activated by other factors leading to an increase in RSNA *via* a positive feedback mechanism. Therefore, it seems plausible that in patients with hypertension increased RSNA is in part the consequence of increased ARNA and an important pathophysiological mechanism for the development of treatment resistant hypertension.

## Cellular Distribution of α2-Adrenoceptors in the Kidney

**Figure 3:**
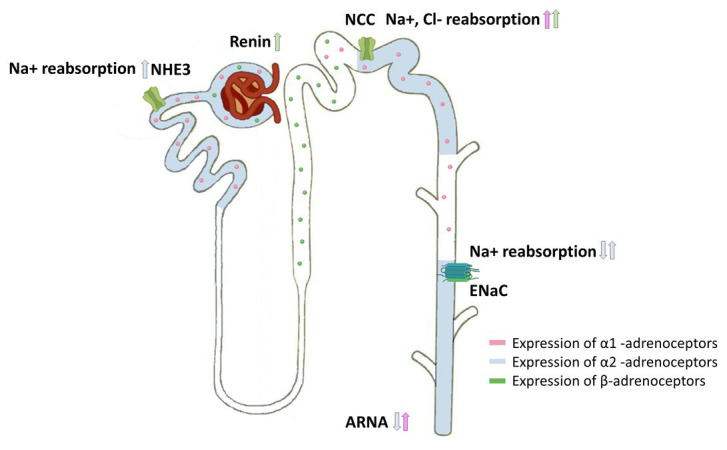
Cellular distribution of adrenoceptors in the nephron and their effects on sodium transporters. Expression of α1-adrenoceptors (pink), α2-adrenoceptors (blue), and β-adrenoceptors (green) along the nephron showing the influence of renal sympathetic neurotransmission on sodium handling. Norepinephrine modulates sodium handling in the proximal (Na+/H+ 1 and 3 Exchange) and distal tubule (NCC and ENaC) by activating α1-adrenoceptors, α2-adrenoceptors, or β-adrenoceptors. Furthermore, α2-adrenoceptor activation within the renal pelvis decreases ARNA, whereas α1-adrenoceptor activation increases ARNA. β-adrenergic receptor activation induces renin release.

There are three different subtypes of α2-adrenoceptors (α2A-, α2B-, and α2C-adrenoceptors; Trendelenburg et al., 2001). The cellular distribution of these subtypes varies, but several *in vivo* and *in vitro* studies confirmed that the α2A-adrenoceptor is the predominant subtype involved in the regulation of renal and cardiac sympathetic neurotransmitter release (Hein et al., 1999; Vonend et al., 2007; Hoch et al., 2011). Based on early results from radioligand binding studies which were confirmed and expanded by deep sequencing analysis of microdissected rat renal tubules (Muntz et al., 1986; Nord et al., 1987; Lee et al., 2015), the cellular distribution of α2-adrenoceptor subtypes along the nephron is now well-described and summarized in Figure 3 and Table 1. α2B-adrenoceptors are expressed in the proximal tubule whereas the α2A-adrenoceptors are located on the connecting tubule, collecting duct and the renal pelvis (Dibona and Kopp, 1997; Kopp et al., 2007; Lee et al., 2015; Table 1). In the glomerulus, only α2B-adrenoceptors seem to be expressed. However, the exact cellular localization is not known (Lee et al., 2015). In VSMCs, all three subtypes are expressed and involved in maintaining vascular tone. However, the distribution of the α2-adrenoceptor subtypes varies based on vascular bed and size of the vessels and species. High amount of α2A-adrenoceptor is expressed in large arteries like the aorta, whereas α2B-adrenoceptor is mostly distributed in small arteries and veins contributing to vasoconstriction (Faber et al., 2001; Kanagy, 2005). In addition, most immune cells express α2-adrenoceptors, with α2A- and α2B-adrenoceptors being the predominant subtypes. Thus, α2A- and α2B-adrenoceptors were detected on macrophages, T-cells, and natural killer cells from rodents and humans (Elenkov et al., 2000; Flierl et al., 2007; Scanzano and Cosentino, 2015; Harwani, 2018).

**Table 1:** Gene expression levels of α1-, α2-, and β-adrenoceptor subtypes along a microdissected rat nephron are summarized.

	ADRA1			ADRA2			ADRB		
	*A*	*B*	*D*	*A*	*B*	*C*	1	2	3
Glomerulus		+			+		+++	+	
Proximal tubule		+			+++				
Henle/thick ascending limb							+++		
Distal convoluted tubule							+++		
Connecting tubule			#	+			++		
Cortical collecting duct			++	+					
Outer medullary collecting duct			+						
Inner medullary collecting duct				++					

Values are expressed as median reads per kilobase million (RPKM) with (+) being the lowest expression and (+++) being the highest expression of adrenoceptors (adapted from Lee et al., 2015). In a functional study, α1D-adrenoceptor expression (#) was found in non-microdissected renal cortex mRNA and linked to NCC regulation (Frame et al., 2019). +, represents lowest expression; +++, represents highest expression.

Taken together, α2-adrenoceptor subtypes are expressed along the nephron, on VSMC and on immune cells. When examining the physiological relevance of α2-adrenoceptors, one has to consider, that physiological activity of α2-adrenoceptors is at least in part dependent on their density (Duzic et al., 1992).

## Effects of α2-Adrenoceptors in the Kidney

The effects of α2-adrenoceptor function in renal physiology can be divided into two categories. First, prejunctional α2A- and α2C-adrenoceptors regulate renal sympathetic neurotransmitter release *via* an autoinhibitory feedback mechanism (Philipp et al., 2002). Activation of prejunctional α-adrenoceptors regulates not only the release of norepinephrine but also the release of ATP, neuropeptide Y (NPY; Burnstock, 1996; Lundberg, 1996; Oberhauser et al., 1999), and thereby modifies renin release, vascular tone, water and sodium handling, as well as the development of renal inflammation and fibrosis by activating different receptors (Dibona and Kopp, 1997; Amann et al., 2000; Bradley et al., 2003; Mcdonough, 2010; Sumi et al., 2010; Kim and Padanilam, 2013).

Second, norepinephrine (in part prejunctional released) activates α2-adrenoceptors expressed on non-adrenergic cells such as renal epithelial cells, VSMCs, or immune cells. This α2-adrenoceptor activation modifies vascular tone, sodium handling, tubulo-interstitial fibrosis, and inflammation within the kidney (Starke et al., 1975; Gilsbach et al., 2009, 2011; Hoch et al., 2011; Kim and Padanilam, 2013; Jang et al., 2019; Hering et al., 2020; Figure 2).

Although the effects of prejunctional released ATP and NPY are not the focus of this review, their effects on renal physiological and pathophysiological mechanisms are important. NPY and ATP, released from sympathetic neurons upon α1- and α2-adrenoceptor signaling (Bradley et al., 2003; Vonend et al., 2007; Sumi et al., 2010), are described to play a role in hypertension (Thulin and Erlinge, 1995) and renal failure (Bald et al., 1997). Additionally to the pleiotropic effects of ATP on its P2 (purinergic type 2) receptors in the kidney (Solini et al., 2015), ATP can also be sequentially hydrolyzed by CD93 to ADP and AMP with AMP being further converted to adenosine by CD73. Alterations in the balance of nucleotides to nucleosides have major impacts on renal function, the development of hypertension, renal fibrosis, and inflammation (for a better overview, please refer to Kishore et al., 2018; Perry et al., 2019).

### α2-Adrenoceptors in Renal Vasculature

Norepinephrine induced renal pressor response is predominantly mediated by α1-adrenoceptors. However, subpressor concentrations of Ang II revealed a role of α2-adrenoceptors in the renal vasoconstrictor response to norepinephrine (Bohmann et al., 1995). Moreover, α2-adrenoceptor activation potentiates Ang II-induced renal pressor response *in vivo* and *in vitro* of SHRs mainly through an α2-adrenoceptor-mediated RhoA-dependent mechanism (Jackson et al., 2001, 2005).

Beside a direct effect on renal vascular resistance, activation of both α1- and α2B-adrenoceptors by chronic renal sympathetic overactivity induces a phenotypic switch of VSMC into proliferative VSMC, leading to hypertrophy, renal vascular stiffness, and reduced renal blood flow (Wang et al., 2004; Huhtinen and Scheinin, 2008). This phenotypic switch is mediated by norepinephrine-induced reactive oxygen species *via* p38 MAPK activation (Kalyankrishna and Malik, 2003; Bleeke et al., 2004). However, the exact mechanism of how increased RSNA induces the development of preglomerular arteriolopathy is still not known. As proof that RSNA is involved in regulating renal pressor response, renal denervation reduced renal sympathetic neurotransmission results in a significant decrease of renal vascular resistance and a significant increase of renal plasma flow as well as GFR in SHR compared to non-denervated SHRs (Tomoda et al., 1997).

Summing up, α2- and α1-adrenoceptors are directly involved in renal vasoconstriction and indirectly by causing a phenotypic switch toward proliferative VSMC.

### Effects of Renal Sympathetic Norepinephrine Release and Epithelial α2-Adrenoceptors on Sodium Homeostasis in Hypertension

Changes in renal vascular resistance and renal blood flow have been shown to influence sodium excretion (Sparks et al., 2015). RSNA affects natriuresis in several animal models and patients with resistant hypertension (Katayama et al., 2013; Poss et al., 2015; Hering et al., 2020). Renal sympathetic nerves also innervate renal tubules. As shown in Figure 3, α1-adrenoceptors, α2-adrenoceptors, and β1-adrenergic receptors are expressed along the nephron (Dibona and Kopp, 1997; Lee et al., 2015; Sata et al., 2018; Kiuchi et al., 2019). During chronic Ang II infusion, sodium and volume excretion was significantly reduced in α2A-adrenoceptor deficient mice compared to wildtype mice (Hering et al., 2020). This impaired natriuretic response was in part caused by an increased abundance of the cleaved epithelial sodium channel (ENaC)-alpha and -gamma subtypes, both markers for ENaC activation (Nguyen et al., 2013; Veiras et al., 2020). The role of α2-adrenoceptors in controlling natriuresis is still not fully understood, as it is hard to distinguish between α2-adrenoceptor-mediated effects and effects mediated by an increased renal sympathetic norepinephrine release. In general, it is widely accepted that increased sympathetic norepinephrine release increases ENaC expression and activation (Mansley et al., 2015; Hering et al., 2020). In contrast, renal denervation has been shown to reduce ENaC and aquaporin2 expression in a mouse model of heart failure, suggesting that the amount of renal norepinephrine release is relevant for the regulation of ENaC expression (Zheng et al., 2019). Besides that, several reports demonstrate an interaction between α2-adrenoceptor activation and regulation of ENaC abundance and activation. Thus, α2-adrenoceptor activation inhibits vasopressin-induced cAMP generation (Chabardes et al., 1984; Krothapalli and Suki, 1984), which in turn decreases vasopressin induced ENaC activation (Roos et al., 2013). In contrast, activation of basolateral α2-adrenoceptors on principal cells increases ENaC activity *in vitro* (Mansley et al., 2015). Thus, only selective deletion of α2A-adrenoceptors from the collecting duct will show evidence about the impact of α2-adrenoceptors on ENaC function.

In addition, sympathetic norepinephrine influences the expression and activation of the Na^+^Cl^−^ co-transporter (NCC) and, thereby, sodium excretion in the distal nephron and the development of hypertension. Norepinephrine stimulates NCC expression through an activation of basolateral Kir4.1/Kir5.1 potassium channel *via* beta-adrenergic receptor activation (Duan et al., 2019). Activation of α1-adrenoceptors inhibits the suppression of NCC during high salt intake *via* a WNK/SPAK/OxSR1-dependent signaling pathway in rat kidneys (Frame et al., 2019). Dephosphorylation of NCC by the protein phosphatase 1 can be inhibited through a protein kinase A-dependent activation of the protein phosphatase 1 inhibitor *via* β1-adrenergic receptor activation (Penton et al., 2019).

In proximal tubules, acute increase in sympathetic norepinephrine has been shown to stimulate sodium hydrogen exchanger 1 and 3 and, thereby, sodium reabsorption most likely by activating α2-adrenoceptors (Nord et al., 1987; Leong et al., 2006; Healy et al., 2014; Lee et al., 2015). In this context, short term stimulation of renal nerves has been shown to activate sodium hydrogen exchanger 3 (NHE3)-mediated sodium reabsorption and the intrarenal renin-angiotensin system (RAS). As this effect was blocked by losartan, a selective Ang II type 1 receptor blocker, the authors suggested that this mechanism is in part mediated *via* an intrarenal RAS activation induced by ERSA (Pontes et al., 2015). NHE3 function is negatively correlated to its phosphorylation status at the PKA site (serine 552) that effects subcellular trafficking and, therefore, its activity (Kocinsky et al., 2005). Ang II treatment decreased cAMP/PKA signaling and, therefore, the phosphorylation at serin 552 leading to increased NHE3 activity (Crajoinas et al., 2016). In contrast, increased sympathetic norepinephrine release in long-term Ang II-dependent hypertension suppresses NHE3 abundance (Nguyen et al., 2013; Hering et al., 2020; Veiras et al., 2020). This suppression of NHE3 is a compensatory natriuretic mechanism of the kidney to regulate blood pressure in chronic hypertension and override the stimulatory effect of Ang II on NHE3 (Mcdonough, 2010; Mcdonough and Nguyen, 2015).

This section highlights the effect of renal sympathetic norepinephrine release on renal sodium transport. The amount of prejunctional released norepinephrine regulates sodium homeostasis in the kidney. There is strong evidence that α2-adrenoceptors play a role in regulating ENaC function, whereas α1- and β-adrenoceptors are involved in the regulation of NCC.

## Role of Renal Sympathetic Neurotransmission and α2-Adrenoceptor Signaling on Immune Cell Function in Hypertension

Although renal sympathetic overactivity plays an important role in the progression of hypertensive kidney disease, its role in the development of fibrosis and inflammation leading to CKD is not fully understood (Veelken et al., 2008; Jang et al., 2019). The therapeutic strategy of renal denervation preventing renal failure may also be at least in part due to its protective anti-inflammatory effect attenuating renal inflammation and fibrosis (Veelken et al., 2008; Kim and Padanilam, 2013). Animal studies show robust evidence that the sympathetic nervous system interacts with the immune system and, thereby, modulates the inflammatory response in the target organ, leading to fibrosis und progression of the underlying disease (Andersson and Tracey, 2012; Carnevale et al., 2016).

In lymphoid organs, sympathetic neurons release norepinephrine which has a direct effect on immune cells by modulating T-cell polarization, lymphocyte trafficking, and proliferation as well as cytokine production *via* adrenoceptor activation. Moreover, immune cell trafficking depends also on regional blood flow which is under tight control of the local sympathetic nerve activity (Elenkov et al., 2000). Although all three adrenoceptor subtypes are expressed within the immune system, β-adrenergic receptor-mediated effects are studied most extensively with special interest for the β2-adrenoceptor subtype due to its anti-inflammatory effects (Elenkov et al., 2000). The role of α2-adrenoceptors on immune cells is still not well-examined but comes into the focus of research (Elenkov et al., 2000; Flierl et al., 2007; Kim and Padanilam, 2013).

A very recent study showed that increased sympathetic nerve activity caused by experimental hypertension increases T-cell homing of effector memory T-cells in the bone marrow *via* β2-adrenergic receptor activation (Xiao et al., 2020). When hypertensive stimuli persist, these effector memory T-cells infiltrate into the vasculature and the kidney and release cytokines like interferon gamma (INF-γ), interleukin-17 (IL-17), and tumor necrosis factor alpha (TNF-α), which aggravate hypertension (Madhur et al., 2010). INF-γ, TNF-α, and IL-17 accelerate vascular damage and water reabsorption by affecting different sodium transporters along the distal nephron (Sriramula et al., 2008; Kamat et al., 2015; Wu et al., 2016). In addition, increased RSNA in low-dose Ang II-dependent hypertension activates antigen presenting cells and, subsequently, T-cells infiltrating hypertensive kidneys. Reduction in RSNA by renal denervation significantly reduced T-cell and macrophage infiltration, attenuated renal fibrosis, and improved renal function (Xiao et al., 2015). These results clearly demonstrate that renal sympathetic nerves mediate renal inflammation and T-cell activation in hypertension. However, there is conflicting evidence concerning the distinctive contribution of renal afferent and efferent nerves on the described anti-inflammatory effects of RDN. Xiao et al. (2015) could not find a contribution of afferent nerves on Ang II-induced hypertension and renal inflammation, whereas Banek showed evidence that renal afferent nerves modulate at least in part renal inflammation in DOCA-salt hypertension (Banek et al., 2016, 2019). In another study, performed in global α2A-deficent mice chronically infused with a high dose of Ang II, increased renal sympathetic norepinephrine release impairs renal function and aggravates hypertension as well as renal fibrosis without affecting the amount of infiltrating immune cells (Hering et al., 2020). At first glance, these results seem to be conflicting as several studies have shown that increased RSNA activates a pro-inflammatory immune cell response leading to immune cell infiltration and an aggravation of renal fibrosis and hypertension (Kim and Padanilam, 2013, 2015; Xiao et al., 2015; Banek et al., 2019). However, α2-adrenoceptors also seem to regulate immune cell function, and activation of α2-adrenoceptors seems to induce a pro-inflammatory immune response (Scanzano and Cosentino, 2015). Thus, inhibition of α2-adrenoceptor on alveolar macrophages reduces the release of several cytokines like TNF-α, IL-6, or IL-1β (Flierl et al., 2007), whereas activation of α2-adrenoceptors on macrophages has been shown to increase TNF-α production (Spengler et al., 1994). In addition, inhibition of renal a2-adrenoceptors reduces renal inflammation and infiltration of neutrophils and macrophages in obstructed murine kidneys, whereas direct infusion of norepinephrine in denervated kidneys induced a fibrotic response similar to innervated non-infused kidneys. These results suggest an important role of norepinephrine signaling through renal α2-adrenoceptors in fibrogenesis and mediating inflammation (Kim and Padanilam, 2013). In line with these observations, a recent study could show that α2A-adrenoceptor deficiency reduced lung injury in mice and decreased lung inflammation by reducing immune cell infiltration as well as decreasing pro-inflammatory cytokines (Cong et al., 2020).

Activation of α1- and α2-adrenoceptors seems to induce polarization toward the inflammatory M1 phenotype (Grisanti et al., 2011; Harwani, 2018), and activated macrophages have been shown to accelerate hypertension (Wenzel et al., 2011). In contrast to the innate immune system, adrenoceptor functions on T-cells are less well described and conflicting. On one side, activation of β2-adrenergic receptors activates the homing of CD8^+^ effector memory T-cells and an upregulation of CCL19 and CCL21 in hypertension. On the other side, activation of β2-adrenergic receptors in experimental autoimmune disease or *in vitro* reduces the T-cell response to sympathetic norepinephrine and decreases the release of INF-γ and TNF-α from CD8^+^ T-cells (Estrada et al., 2016; Araujo et al., 2019). Although α2-adrenoceptors are expressed on T-cells, their role in T-cell function in general and particularly in hypertension is not well understood. Early studies have shown that reduced peripheral blood T-cell mitogenesis is caused by activation of peripheral α2-adrenoceptors (Felsner et al., 1995). Activation of α2-adrenoceptors expressed on dendritic cells reduces induction of T-cell proliferation (Araujo et al., 2019). In patients undergoing surgery, activation of α2-adrenoceptors shifted the Th1/Th2 and the Treg/Th17 cytokine balance toward a Th1 and Th17 response, respectively, suggesting a pro-inflammatory rather than an anti-inflammatory effect on human T-cells (Lee et al., 2018).

In conclusion, increased sympathetic norepinephrine release seems to activate T-cell response through a complex interaction with the innate immune system leading to an aggravation of hypertension and CKD. The role of adrenergic receptors in modulating the immune response in hypertensive kidney damage needs further investigation but seems to be an interesting therapeutic approach, as selective agonists and antagonists of α- and β-adrenergic receptors are already in clinical practice.

## Conclusion and Limitation

Renal sympathetic nerve activity plays a major role in blood pressure homeostasis. Regulation of RSNA describes a complex interaction between afferent nerve activity and central mechanism. Under physiological conditions, RSNA is controlled by afferent renal mechano- and chemo-sensitive nerves by the renorenal reflex *via* a negative feedback mechanism. Thereby, ARNA is regulated in part by α1- or α2-adrenoceptors located in the renal pelvis. During hypertension or kidney damage, this negative feedback mechanism is disturbed. Activation of afferent renal nerves induced by several factors including uremic toxin, pro-inflammatory cytokines, and hypoxia injury increases RSNA and is therefore an important factor for the development of resistant hypertension and kidney disease.

Increased sympathetic nerve activity results in an elevated release of sympathetic neurotransmitter. Prejunctional α2-adrenoceptors control renal sympathetic neurotransmission *via* a negative feedback mechanism. Deletion or pharmacological inhibition of α2-adrenoceptors accelerates hypertension and kidney injury through multiple mechanisms. First, increased sympathetic neurotransmission particularly norepinephrine release increases renin release, renal vascular tone, sodium reabsorption, and inflammation through an activation of α- and β-adrenoceptors in the kidney and on immune cells. Second, non-adrenergic α2-adrenoceptor activation on renal epithelial cells, VSMCs, or immune cells directly modulates vascular tone, sodium balance, and immune cell response in the kidney. Based on this complex interaction between the well-studied function of prejunctional α2-adrenoceptors and the multiple effects of adrenoceptors activation on non-adrenergic cells in the kidney and on immune cells, the exact physiological and pathophysiological role of α2-adrenoceptor is still not fully understood and needs further studies in where α2-adrenoceptor function can be examined cell specific.

## Author Contributions

All authors listed have made a substantial, direct and intellectual contribution to the work, and approved it for publication.

### Conflict of Interest

The authors declare that the research was conducted in the absence of any commercial or financial relationships that could be construed as a potential conflict of interest.
